# Disturbance Recognition and Collision Detection of Manipulator Based on Momentum Observer

**DOI:** 10.3390/s20154187

**Published:** 2020-07-28

**Authors:** Xiaojun Zhang, Jian Zhao, Minglu Zhang, Xiaoyu Liu

**Affiliations:** School of Mechanical Engineering, Hebei University of Technology, Tianjin 300132, China; zj_lonzo@163.com (J.Z.); zhangml@hebut.edu.cn (M.Z.); lxy_amber@163.com (X.L.)

**Keywords:** nonlinear disturbance, momentum observer, parameter identification, collision detection, human-robot interaction

## Abstract

Increasing requirements for the safety of human-robot interaction and the cost-effectiveness of collision detection rapidly promote the development of collision detection technology without torque sensors. To address nonlinear disturbance factors in collision detection that may cause unstable or even incorrect detection, this paper proposed a research strategy that considered the friction as the disturbance term in manipulator motion for the collision detection. The manipulator joint disturbance model was established based on the LuGre dynamic friction model, and the external torque observer was designed based on the generalized momentum. Then, the friction measurement was realized using the external torque observer, and the model parameters were identified through the genetic algorithm. The collision detection can be reduced errors after the friction model by compensating the disturbance and can be applicable to variable working conditions. Finally, the accuracy of the constructed disturbance model and the performance of the proposed collision detection method were validated by the experimental studies.

## 1. Introduction

With the expansion of robot applications ranging from industrial environments to home services, medical treatments, and space exploration, human-robot collaboration has become a hot topic [1,2]. Compared with industrial robots that can only work in the fence, collaborative robots can share working spaces with humans. The safety of human, environment, and robot body in the unknown environment is worthy of studying in depth [3]. Compared to traditional industrial robots operating in the structured environment, the working environment of collaborative robots is not isolated and unpredictable. The International Organization for Standardization (ISO) has proposed relevant technical standards about collaborative robot safety, including safety specifications, risk assessments, and so on, to minimize the potential risk caused by human-robot collision [4]. Solving safety problems can start with collision detection. When the robot is running in an unstructured environment, uncertainty in the workspace causes the human-robot accidental collision [5]. The collaborative robot with safety and dexterity can detect each joint torque and respond to the collision detection. Optimizing human-robot collision detection in unpredictable environments is the most critical and urgent issue facing collaborative robots. At present, some scholars have made efforts in this area and achieved certain achievements [6,7,8].

The traditional methods applied for the collision detection of collaborative robot often adopt the scheme of installing an external sensor, such as a joint torque sensor [9,10]. Haddadin et al. constructed an external torque observer based on generalized momentum to achieve collision detection on variable robots with joint torque sensors such as DLR LWR-III, KUKA LBR iiwa, and FRANKA EMIKA [11]. They further analyzed the energy characteristics to improve control based on joint torque information and made the robot obtain the skill as a human can dribble blindly [12]. Cho et al. proposed a new collision detection algorithm by observing the speed of external torque changes based on the data of the joint torque sensor. They distinguished expected contact from unintended contact and enabled collision detection to be used in complex tasks such as teaching and playback [13]. The depth camera positioning combining with joint torque information could accurately estimate the collision position and the magnitude of the collision force, which further improve the ability of human-robot cooperation [14]. The installation of a torque sensor could guarantee the safety of human and robot during the collaboration and make collision detection applicable to more complex occasions. However, this method has the disadvantages of increasing the complexity of joint design, limiting the working performance, and increasing the manufacturing cost.

In contrast, the scheme of sensorless collision detection has no effect on the performance of the manipulator because of nothing changed in the structural design. Most of the existing research constructed virtual sensors based on information, such as calculation models and joint currents, to estimate the collision moment of the manipulator [15,16,17,18]. Yen et al. implemented a collision detection scheme for low-cost robots, such as home and service with a controller. However, the sensor signal of the manipulator body is complex, and there is often a phenomenon of false collision detection in the virtual sensor [19]. A band-pass filter that considered the frequency boundary of the dynamic model was designed to extract collision information from the mixed estimated signal and improve the accuracy of the collision detection on the manipulator when the sensor data is uncertain [20]. By improving the filtering characteristics of the virtual sensor, the manipulator could distinguish between contact and collision during human-robot interaction, which provided more information support for collision reaction [21]. Based on deep learning approach, Heo et al. designed a deep neural network model to learn robot collision signals and accepted high-dimensional signals from robot joints as input to achieve high sensitivity to collisions and low susceptibility to false alarms [22].

Sensorless collision detection usually adopts joint current information to estimate joint torque, but there is a nonnegligible nonlinear error with the actual joint torque. So, it is a major obstacle to the improvement of the sensorless collision detection performance of the manipulator. Nonlinear errors are mainly caused by internal and external disturbances, including joint friction, assembly clearance, temperature, and other factors, where friction is the main component of nonlinear errors [23]. Most studies have identified friction parameters based on Coulomb and Viscous friction during the parameter identification of the dynamic model, which can effectively reduce the influence of nonlinear errors [24,25]. However, the working conditions of the manipulator are complicated and variable, and these methods fail to deal with other nonlinear factors. Solving this defect, another research channel is to compensate the error of nonlinear factors based on the control method [26]. Xu et al., based on the friction observer, compensated the estimated friction torque through the joint controller [27]. However, the control method compensated friction mainly via position control and speed control. When the collision happened, the friction force and the collision force could not be well distinguished.

In variable working environments of the manipulator, the nonlinear factors change accordingly based on temperature, friction, load and so on. To reduce the workload caused by the processing of the nonlinear factors, the corresponding disturbance model can be directly transformed under different operating conditions without completely abandoning the original workload. In this paper, a method of disturbance recognition and collision detection for the manipulator based on an external torque observer was proposed. In the laboratory environment, the friction was considered as the main disturbance factor. The second-order external torque observer based on generalized momentum was constructed to observe the disturbance torque. The disturbance model based on the LuGre dynamic friction model was established to compensate the disturbance term after identifying its parameters through the genetic algorithm. To verify the performance of the proposed method, three collision detection experiments were carried out based on a modular collaborative manipulator platform. The experimental analysis results show that the proposed method can quickly and accurately observe the change of the external joint torque and realize the collision detection.

The rest of the article is organized as follows. In Section 2, the derivation of the manipulator dynamic model is introduced, and the construction of the second-order external torque observer is described in detail. Establishment of the LuGre friction model, observation of the friction torque based on the observer, and identification of the model parameters by the genetic algorithm are presented in Section 3. Section 4 sets up the collision detection experiments based on the physical manipulator and analyzes the verification results. Section 5 provides some conclusions drawn from this research.

## 2. Theoretical Analysis and Model Foundation

### 2.1. Manipulator Dynamics Modeling

When collisions between the manipulator and the outside occur, it is equivalent to the external force acting on the collision point, which generates additional torque on each manipulator joint. The premise of accurately analyzing the collision moment of the manipulator is to establish a precise dynamic model [28,29].

Based on the improved D-H coordinate system, the Newton–Euler recursion formula is used to establish the manipulator dynamics equilibrium equation. The manipulator dynamics model can be expressed as Equation (1):

(1a) $$M(q)\ddot{q}+C(q,\dot{q})\dot{q}+G(q)=K_{J}(\theta -q)+D_{J}(\dot{\theta }-\dot{q})-\tau_{F,q}$$

(1b) $$B\ddot{\theta }+K_{J}(\theta -q)=\tau_{m}-D_{J}(\dot{\theta }-\dot{q})-\tau_{F,\theta }$$

where $M(q)\in R^{n\times n}$ represents the symmetrical positive definite inertia matrix of the manipulator link, $C(q,\dot{q})\in R^{n}$ contains the centrifugal moment and Coriolis moment vector of the manipulator link, $G(q)\in R^{n}$ represents the moment of gravity, B is the motor inertia matrix, $K_{J}$ is the joint stiffness matrix, $D_{J}$ is the joint damping matrix, $\tau_{F,q}$ is the friction torque of the manipulator link, $\tau_{F,\theta }$ is the friction torque on the motor side, $\tau_{m}$ is the output torque of the motor, q indicates the position of the manipulator link, θ indicates the motor position, and $\dot{q}$ indicates the movement speed of the manipulator.

In addition, the output torque of the joint can be defined as $\tau_{J}$. Set up $\tau_{J}=K_{J}(\theta -q)$ and ignore the joint damping term $D_{J}$ [11]. Therefore, the dynamic model of the manipulator Equation (1) can be simplified as Equation (2):

(2a) $$M(q)\ddot{q}+C(q,\dot{q})\dot{q}+G(q)=\tau_{J}-\tau_{F,q}$$

(2b) $$B\ddot{\theta }+\tau_{J}=\tau_{m}-\tau_{F,\theta }$$

When the manipulator collision occurs, the joint torque increases. So, the dynamic model can be modified by Equation (3):

(3a) $$M(q)\ddot{q}+C(q,\dot{q})\dot{q}+G(q)=\tau_{J}-\tau_{F,q}-\tau_{ext}$$

(3b) $$B\ddot{\theta }+\tau_{J}=\tau_{m}-\tau_{F,\theta }$$

where $\tau_{ext}$ is the external torque of the collision.

### 2.2. External Torque Observer Design

Haddadin et al. proposed an external torque observer based on generalized momentum to detect the collision [11]. In this paper, the disturbance force was classified as an external force. An external torque observer based on generalized momentum was constructed by referring to the observer design. When no collision occurs, this observer was a disturbance observer in fact. Since the friction force was the main part of the disturbance force, the friction torque of each joint can be observed through the disturbance observer to avoid the introduction of joint angular acceleration $\ddot{q}$.

The generalized total momentum $p_{tot}$ of the manipulator system can be defined as Equation (4):

(4) $$p_{tot}=p_{q}+p_{\theta }=M(q)\dot{q}+B\dot{\theta }$$

where $p_{q}$ is link momentum, and $p_{\theta }$ is motor momentum. Derivation of Equation (4) to time t yields Equation (5):

(5) $${\dot{p}}_{tot}={\dot{p}}_{q}+{\dot{p}}_{\theta }=M(q)\ddot{q}+\dot{M}(q)\dot{q}+B\ddot{\theta }$$

According to the basic property of oblique symmetry in manipulator dynamics: $\dot{M}(q)-2C(q,\dot{q})$, it can also be expressed as $\dot{M}(q)=C(q,\dot{q})+C^{T}(q,\dot{q})$. Simplify Equation (5) to get Equation (6):

(6a) $${\dot{p}}_{tot}=\tau_{m}-\beta (q,\dot{q})-\tau_{F,tot}$$

(6b) $$\beta (q,\dot{q})=G(q)-C^{T}(q,\dot{q})\dot{q}$$

(6c) $$\tau_{F,tot}=\tau_{F,q}+\tau_{F,\theta }+\tau_{ext}$$

(6d) $$\tau_{m}=K_{I}\times i$$

where $K_{I}$ represents the product of the torque constant of the joint motor and the reduction ratio of the joint reducer, and i is the motor output current value, ignoring the torque caused by the motor inertia $B\ddot{\theta }$.

When no collision occurs with the manipulator, $\tau_{ext}=0$. The above $\tau_{F,tot}$ is expressed as $\tau_{F,tot}=\tau_{F,q}+\tau_{F,\theta }$.

In order to meet the requirements of fastness and stability, and avoid the shortage of the first-order system with few adjustment parameters, the first-order observer was optimally designed to construct a second-order external torque observer, and its dynamic model was modified as Equation (7):

(7a) $${\dot{\hat{p}}}_{tot}=\tau_{m}-\beta (q,\dot{q})-r_{F}$$

(7b) $${\dot{r}}_{F}=K_{1}({\dot{p}}_{tot}-{\dot{\hat{p}}}_{tot})-K_{2}r_{F}+K_{1}K_{2}\int_{0}^{t}r_{F}dt$$

where $K_{1},K_{2}$ are the diagonal gain matrix. External torque observer $r_{F}$ can be expressed as Equation (8):

(8) $$r_{F}=K_{1}(p(t)-\int_{0}^{t}(\tau_{m}-\beta (q,\dot{q})-r_{F})dt-p(0))-K_{2}\int_{0}^{t}\left(r_{F}-\int_{0}^{t}K_{1}r_{F}dt\right)dt$$

Derivation of Equation (8) to time t: ${\ddot{r}}_{F}=K_{1}{\dot{\tau }}_{F,tot}-K_{1}{\dot{r}}_{F}-K_{2}{\dot{r}}_{F}+K_{1}K_{2}r_{F}$.

According to the Laplace transform, the second-order observer can be represented by the multiplication of a first-order low-pass filtering and a first-order high-pass filtering, and Equation (9) can be drawn as follows:

(9) $$\frac{r_{F,i}}{\tau_{F,tot,i}}=\frac{K_{1,i}s}{s^{2}+\left(K_{1,i}+K_{2,i}\right)s+K_{1,i}K_{2,i}}=\frac{K_{1,i}}{s+K_{1,i}}\cdot \frac{s}{s+K_{2,i}}\left(i=1,\ldots ,n\right)$$

The low-pass and high-pass filter was appropriately designed to analyze the collision frequency, including high-frequency torque components of fast and strong impacts and low-frequency torque components of slow and continuous contacts. Considering that there may be a large overshoot and steady-state error in the second-order external torque observer [30,31], while reducing the detection delay and oscillation, the control algorithm was added to the second-order external torque observer, and the improved observer was defined as Equation (10):

(10) $$\begin{array}{l} r_{F}=K_{1}(p(t)-\int_{0}^{t}(\tau_{m}-\beta (q,\dot{q})-r_{F})dt-p(0))-K_{2}\int_{0}^{t}\left(r_{F}-\int_{0}^{t}K_{1}r_{F}dt\right)dt\\ +K_{3}\left(p(t)-\int_{0}^{t}\left[\tau_{m}-\beta (q,\dot{q})\right]dt-p(0)\right) \end{array}$$

where $K_{3}$ is the diagonal gain matrix.

According to the Laplace transform, Equation (11) can be drawn as follows:

(11) $$\frac{r_{F,i}}{\tau_{F,tot,i}}=\frac{\left(K_{1,i}+K_{3,i}\right)s}{s^{2}+\left(K_{1,i}+K_{2,i}\right)s+K_{1,i}K_{2,i}}\left(i=1,\ldots ,n\right)$$

**Figure 1:**
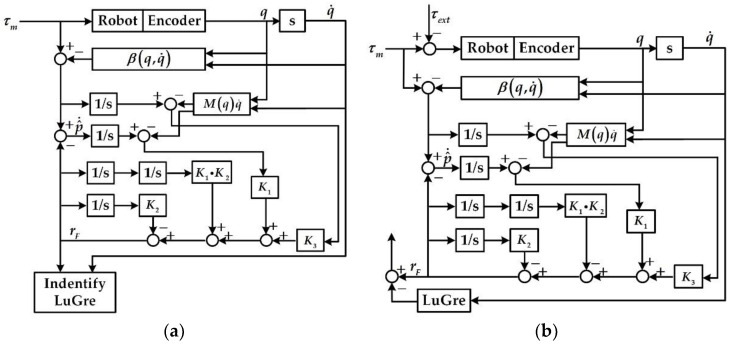
Block diagram of proposed estimation method architecture: (**a**) Identification of the LuGre model; (**b**) Observation of the external torque.

To detail the logical architecture of proposed estimation method, the process of using the external torque observer to identify the LuGre model and obtain the observed external torque after the LuGre model compensation is shown in Figure 1.

## 3. Friction Modeling and Parameter Identification

Friction models mainly include static models such as Coulomb-Viscous friction model and Stribeck model, and dynamic models such as Dahl and LuGre. The LuGre model is based on the average deformation of the bristle, using first-order differential equations to describe Coulomb friction, viscous friction, Stribeck friction, presliding friction, variable static friction, friction hysteresis, and so on [32,33]. The static characteristics and dynamic characteristics of friction can be well described by the LuGre model. It is a dynamic friction model that is relatively complete and easy to implement.

The accuracy of the friction model significantly affects the performance of the manipulator. In this paper, the LuGre model was selected to describe the manipulator joint friction. This exponential model can greatly reflect the nonlinear characteristics of friction. Since the motion type of each manipulator joint is rotation, the friction torque $\tau_{f,tot}$ can be expressed by Equation (12):

(12a) $$\tau_{f,tot}=\sigma_{0}z+\sigma_{1}\frac{dz}{dt}+\sigma_{2}\omega$$

(12b) $$\frac{dz}{dt}=\omega -\frac{\sigma_{0}}{s(\omega )}z\left|\omega \right|$$

(12c) $$s(\omega )=\tau_{c}+(\tau_{s}-\tau_{c})e^{-{\left(\omega /\omega_{s}\right)}^{2}}$$

where z is the state variable representing the average deformation of the bristle, ω is the rotation angular velocity, $\sigma_{0}$ is the bristle stiffness coefficient, $\sigma_{1}$ is the microdamping coefficient; $\sigma_{2}$ is the viscous friction coefficient, $\tau_{c}$ is the Coulomb friction, $\tau_{s}$ is the static friction, and $\omega_{s}$ is the Stribeck speed.

The accuracy of identification parameters in the friction model largely determines the credibility of solving practical problems based on the model. Therefore, it is necessary to effectively identify the friction model parameters based on an appropriate identification algorithm [34,35]. In this paper, the genetic algorithm with strong parallel iteration ability wa selected to identify parameters in the friction model. There are six unknown parameters $\left(\sigma_{0},\sigma_{1},\sigma_{2},\tau_{c},\tau_{s},\omega_{s}\right)$ in Equation (12) to be identified.

To facilitate the identification of model parameters, that is, to identify the above six dynamic and static parameters at once, the LuGre friction model was improved and discretized. The microdisplacement z and microvelocity $\frac{dz}{dt}$ were used as the intermediate variables of the system to simplify the model and avoid measuring. Assuming that the discretized sampling time interval is ΔT and the discretized time is k, the recursive formula of the discretized LuGre friction model can be drawn as Equation (13):

(13a) $$s(k)=\tau_{c}+(\tau_{s}-\tau_{c})e^{-{\left(\omega (k)/\omega_{s}\right)}^{2}}$$

(13b) $$\dot{z}(k)=\frac{\omega (k)-\frac{\sigma_{0}}{s(k)}z(k-1)\left|\omega (k)\right|}{1+\frac{\sigma_{0}}{s(k)}\Delta T\left|\omega (k)\right|}$$

(13c) $$z(k)=\sum_{i=0}^{k}\dot{z}(i)\Delta T$$

(13d) $$\tau_{f,tot}(k)=\sigma_{0}z(k)+\sigma_{1}\dot{z}(k)+\sigma_{2}\omega (k)$$

Because the LuGre friction model is highly nonlinear and has first-order differential terms, it is easy to fall into the local optimal problem during the process of the model parameter identification by genetic algorithm [36]. In response to the above, different initial rounds of evolutionary optimization initial parent populations were set up to solve the local optimal problem of parameter identification for highly nonlinear differential LuGre friction model.

Let the error e of friction torque as Equation (14):

(14) $$e(k)=\tau_{F,tot}-\tau_{f,tot}$$

where $\tau_{F,tot}$ is the observation of friction torque based on the designed observer, and $\tau_{f,tot}$ is the calculation of friction based on the LuGre friction model.

Define the objective function of the genetic algorithm as Equation (15):

(15) $$J=\frac{1}{2}\sum_{i=1}^{N}e{\left(i\right)}^{2}$$

where N is the sampling times. The goal of identification is to minimize the objective function J.

This study supposed that the manipulator moves in a sinusoidal trajectory as y=0.6sin(6.28*0.2*t), with the time interval of 0.008 s and obtains a total of 1251 samples. The genetic algorithm parameters were set as follows: The initial population was 200, the evolution generation was 300, the selection crossover probability was 0.8, and the mutation probability was 0.2. Besides, the range of parameters to be identified was set up as follows: $\sigma_{0}\in \left(60000,100000\right]$, $\sigma_{1}\in \left(0,500\right]$, $\sigma_{2}\in \left(0,10\right]$, $\tau_{c}\in \left(0,10\right]$, $\tau_{s}\in \left(0,10\right]$, $\omega_{s}\in \left(0,0.1\right]$.

The parameter identification of the three-joint manipulator by the genetic algorithm was performed. Since the initial speed and the end speed of the planned sinusoidal trajectory were not 0, in order to avoid the impact of data errors during start or stop and protect the manipulator, the fifth-degree polynomial curve was used to connect the sinusoidal trajectory. Make the manipulator run for several cycles and obtain the observation data in the middle stable operation cycle to identify the friction model parameters. Take the average of 10-time identification results, as shown in Table 1.

The six unknown parameters in the discretized LuGre model were identified by the genetic algorithm. Moreover, the trouble with partial effectiveness of linear identification in the two-step identification method was avoided, and the accuracy of parameter identification was improved. Based on the identified model parameters in Table 1, the joint friction torque can be calculated by the LuGre model.

**Figure 2:**
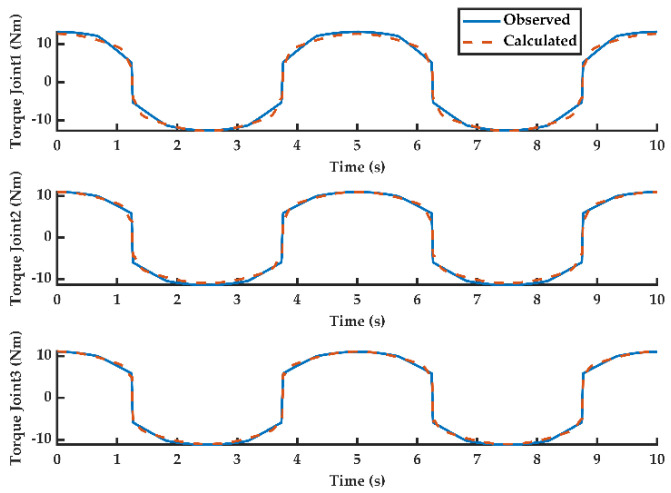
Calculated friction joint torque and observed disturbance.

Observation of the disturbance based on the designed observer and calculation of friction based on the identified model are shown in Figure 2. It can be drawn that the results of the disturbance observation are almost consistent with the results of the friction calculation. In this study, the calculation results are also further verified.

The friction is complex and variable, and it is highly related to the joint speed direction. It is difficult to extract the friction from the joint torque. A reliable method for the above problem was proposed by the authors of [37]. Considering that the speed direction is independent of the calculated joint torque by dynamics model, the two types of trajectories are characterized by the same position, acceleration, and speed value, and the opposite speed direction. Subtracting the joint torque values of these two trajectories obtains the doubled friction values. According to the above procedure, the friction during the sinusoidal trajectory performed by the manipulator is extracted from the output motor torque $\tau_{m}$. To quantify the error between the calculated friction value and the actual friction value, the RMS (root mean squared) error is calculated in Table 2.

Considering other nonlinear factors, the RMS error is a bit large. However, it is still in the within the range of theoretical error, the accuracy of model identification results is proved.

According to the LuGre friction model obtained by the identification, the friction of each operating manipulator joint can be calculated in real time and be employed to compensate the external torque observed by the external torque observer. The purposes of this strategy are to decrease the error caused by the friction and other disturbances in the external torque and raise the precision of the external torque observer enough to make external torque observer applicable to collision detection.

## 4. Collision Detection and Experiment Validation

**Figure 3:**
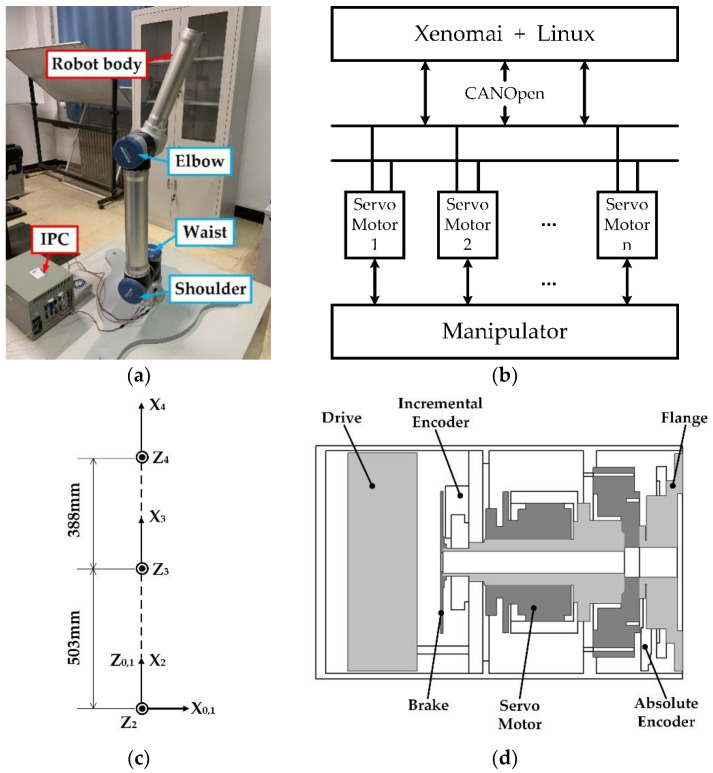
The structure of the test platform and control system: (**a**) Prototype of the manipulator; (**b**) Scheme of control communication; (**c**) Parameters of links; (**d**) Diagram of encoder position.

In this paper, the experiments based on the modular light cooperative manipulator developed independently by the laboratory have been conducted. The experiment platform was composed of a manipulator body and an industrial control computer. The industrial computer used the Xenomai core to expand Linux [38,39], which can meet the real-time control requirements of the manipulator and communicate with the servo driver through the CAN bus. The structure of the experimental platform and control system is given in Figure 3a,b. The size parameters of manipulator links are given in Figure 3c. The input side of each manipulator joint was equipped with the incremental encoder, and the output side was equipped with the absolute encoder, with position of the encoders shown in Figure 3d. The absolute encoder was characterized by 19-bit single-turn with the resolution 0.0007 and the repeated positioning accuracy of 0.001°, and the incremental encoder was characterized by 2500 pulses with the resolution of 10000.

The parameters of position and speed of the joint were required for the external torque observer designed in our research. They were measured by a high-precision absolute value encoder and an incremental encoder, respectively. After compensation by the LuGre friction model, the external torque changes of the manipulator could be observed in real time. However, due to the disturbance term concluding the friction and other nonlinear factors, a few errors existed between the value observed by the external torque observer and the actual external torque value. In the collision detection experiment, this could be handled by setting a threshold to avoid false detection.

According to standards in ISO/TS 15066:2016, since the manipulator body shapes with a cylindrical design in that a large contact area when a collision occurs, the actual collision pressure is much smaller than the maximum allowable pressure, so the actual collision force becomes the main consideration. When the manipulator makes the operation occur without disturbance for a long time [0,T], the maximum value of the observed external torque by the observer is set as a threshold, defined as $\mu_{\max}=\max\{\left|\mu (t)\right|,t\in [0,T]\}$, where μ(t) is the observed external torque after the LuGre model compensation. Considering the stability of collision detection, the threshold adding a small safety margin $\varepsilon_{safe}>0$ is converted to $\varepsilon =\mu_{\max}+\varepsilon_{safe}$. ε=7.80 N⋅m is selected as a reasonable threshold determined by six hours of experiment observation. The threshold range satisfies the human injury threshold recommended in ISO/TS 12066-2016. The occurrence of collision detection is judged by the function of threshold collision detection $f\left(\mu \left(t\right)\right)=\{\begin{array}{ll} 1, & \left|\mu \left(t\right)\right|>\varepsilon \\ 0, & \left|\mu \left(t\right)\right|<\varepsilon \end{array}$, where the i link is involved in a collision, determining the elements in the fault signature matrix η by the above function, the isolation of collision link is achieved by $\eta ={\left[\begin{array}{cc} \underset{\begin{array}{cc} \begin{array}{cc} 1 & ∼ \end{array} & i \end{array}}{\underset{︸}{\begin{array}{cc} 1 & \begin{array}{cc} ,\cdots , & 1 \end{array}, \end{array}}} & \underset{\begin{array}{ccc} i+1 & ∼ & n \end{array}}{\underset{︸}{\begin{array}{ccc} 0 & ,\cdots , & 0 \end{array}}} \end{array}\right]}^{T}$.

In order to verify the effectiveness of the disturbance recognition method based on the external torque observer and the performance of the collision detection between the manipulator and the outside, it is assumed that the task of the manipulator is to grab the workpiece located at the lower left of the manipulator. When working, we consciously touch its links from all direction to simulate the possible collision during the process of actual human-robot collaboration. In the human-robot collision, there is no pain in the human body. Since the average pain tolerance of the human arm is 150~160 N, the contact force is guaranteed to be less than 150 N. So, the intensity of the collision is in accordance with the safe collision amplitude specified in ISO/TS 15066:2016.

**Figure 4:**
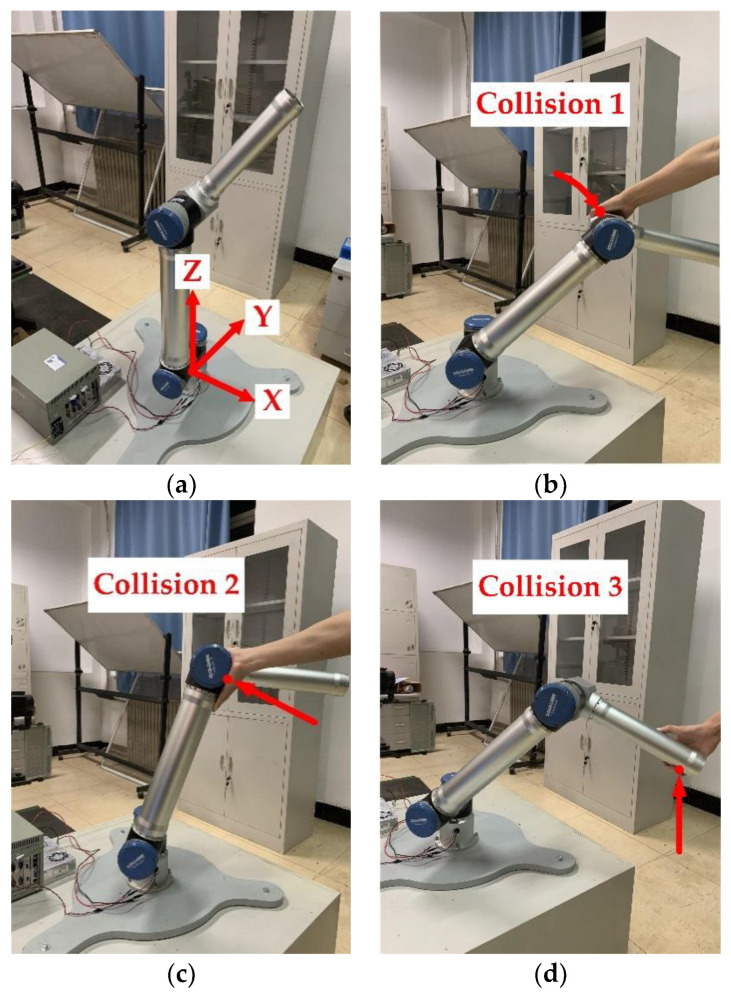
Experimental verification of collision detection performance: (**a**) Initial posture; (**b**) First experiment: Collision 1 in the waist joint; (**c**) Second experiment: Collision 2 in the shoulder joint; (**d**) Third experiment: Collision 3 in the elbow joint.

In this paper, multiple collision experiments in different directions were arranged, and a total of three different collision experiments were described in detail. The manipulator moved from the initial pose shown in Figure 4a. The first experiment was a waist joint collision, the second experiment was a shoulder joint collision, and the third experiment was an elbow joint collision. The test plan and experiment effect are illustrated in Figure 4b–d.

**Figure 5:**
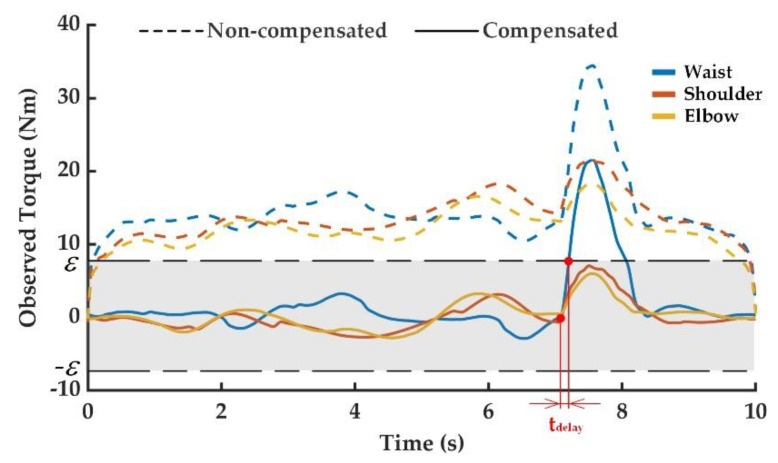
External torque observation in the first collision experiment.

**Figure 6:**
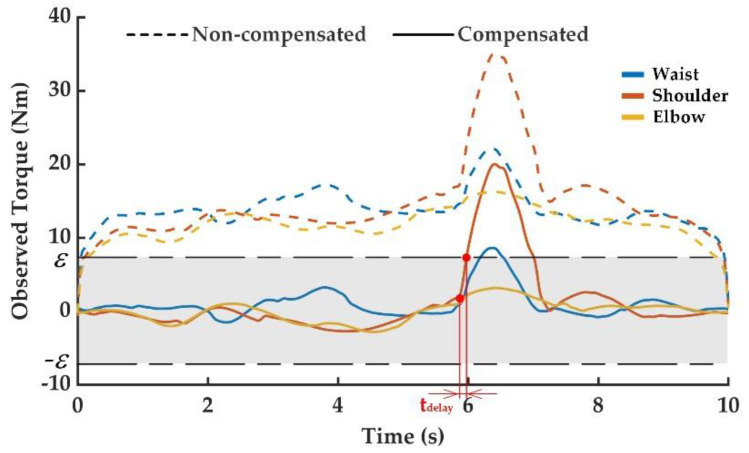
External torque observation in the second collision experiment.

**Figure 7:**
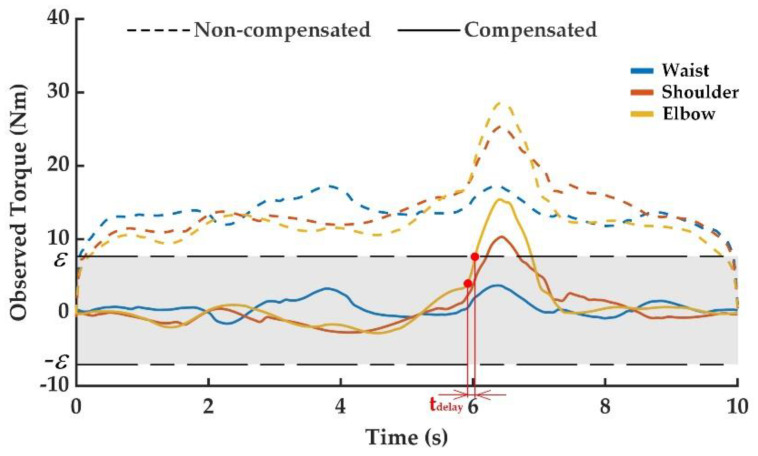
External torque observation in the third collision experiment.

After colliding during the operation of the manipulator, the joint torque increased dramatically. The external torque observation value suddenly changed and was obviously different from the normal observation value. Therefore, the occurrence of the manipulator collision could be determined by the external torque observation value exceeding the threshold. The observed external torque curves of the above collision experiments are shown in Figure 5, Figure 6 and Figure 7, including the one without friction compensation and the one after friction compensation. Through the comparison of results in Figure 5, Figure 6 and Figure 7, it can be seen that the deviation of the observed external torque was large without friction compensation, but the deviation after friction compensation was significantly reduced. Friction compensation could effectively shrink the collision detection threshold and improve the detection sensitivity of the proposed collision detection methodology.

In the first collision experiment as shown in Figure 4b, the collision direction was set to be perpendicular to the plane composed of the second and third manipulator links, and the collision point was set at the end of the second link. This experiment was performed to simulate the human-robot collision caused by the movement of the waist joint driving the manipulator to grab the workpiece in the horizontal plane. The results, as shown in Figure 5, demonstrate that the most obvious mutation existed in the external torque observation value of the waist joint. When the external torque observation value increased until exceeding the set threshold of 7.8 N, the occurrence of waist joint collision could be determined. The happened when the collision time was 7.104 s, actual detected collision time was 7.184 s, and detection delay time was about 0.08 s. When the collision contact occurred, there was the effect of the friction between the human body and collision area. At this time, the continuing movements of the shoulder and elbow joints were prevented by the friction, so the torque change was also observed in the shoulder and elbow joints during the collision. The observation value of the external torque is largely consistent with the theoretical analysis value.

In the second collision experiment as shown in Figure 4c, the collision point was set at the second link of the manipulator and the collision direction was set to be the tangent direction of the shoulder joint motion to prevent moving downward. This experiment was performed to simulate the human-robot collision caused by the movement of the shoulder joint driving the manipulator to grab the workpiece in the vertical plane. It can be seen from Figure 6 that the collision had the greatest impact on the shoulder joint. When the external torque observation value of shoulder joint increased until exceeding the set threshold of 7.8 N, the occurrence of shoulder joint collision could be determined. The happened when the collision time was 5.872 s, actual detected collision time was 5.960 s, and detection delay time was 0.088 s. Due to a certain distance in space between the direction of the collision and the axis of the waist joint, a moment was generated to hinder the movement of the waist joint and affected its torque observation value. The impact on the elbow joint was the smallest due to the lack of contact with the third link.

In the third collision experiment as shown in Figure 4d, the collision point was set at the end of the manipulator and the collision direction was perpendicular to the ground opposite to the movement direction of the manipulator. This experiment was performed to simulate the collision caused by the end effector of the manipulator during the movement. It can be obtained from Figure 7 that the external torque changes of the elbow joint and the shoulder joint were the clearest, because the collision directly hindered the movement of them and had the greatest impact on them. When the external torque observation value of elbow joint increased until exceeding the set threshold of 7.8 N, the occurrence of elbow joint collision could be determined. The happened when the collision time was 5.912 s, actual detected collision time was 6.008 s, and detection delay time was about 0.096 s. Due to the direction of the collision parallel to the axis of the waist joint, the impact on the waist joint was the smallest.

The experiment results illustrate that the external torque observation value fluctuated slightly around 0 while the manipulator operated normally, and the external torque observation value responded to abrupt changes quickly while the collision occurred. Through the simulation of different collision schemes, it can be drawn that the sudden change of the observed values is consistent with the theoretical analysis of the actual manipulator torque. Also, the fluctuation of external torque observation after friction compensation is significantly different from the collision torque, which can effectively detect the occurrence of the manipulator collision.

In order to verify the universal applicability and repeatability of the proposed collision detection method, 50 collision experiments were conducted with random collision direction in space. The collision points were randomly set at the second or third manipulator link. The experiment results have shown that the occurrence of collisions can be detected by the designed procedure, and the success rate of collision detection was 100%. In these collision experiments, the longest detection delay time was 0.096 s and the shortest was 0.064 s. With the manipulator colliding, the detected external torque was all less than 8 N, which ensures the sensitivity of collision detection.

Besides, this research scheme only requires collecting the information of the position, velocity, and current of each manipulator joint, which can avoid the influence of acceleration noise on external torque observation. This solution can be applied to detect the occurrence of collisions on most manipulators with current feedback. To ensure the safety of human-robot collaboration and reduce the risk caused by human-robot collision, it is necessary to make the manipulator switch current motion mode while detecting the collision and take it get out of the collision area. The simplest safe strategy is to stop the manipulator from moving, but it cannot leave the collision area when the squeeze collision occurs in the manipulator with human. Another solution is to take the manipulator reverse action and escape the collision area. This solution requires the construction of a fault signature matrix to locate the accurate collision isolation, and inaccurate position judgment may make the reverse action possible to cause secondary human damage. The safer method is to transfer the manipulator into zero-gravity mode. On this occasion, the servo controller is in the torque-control mode, the gravity and friction are overcome by the joint output torque, and the flexible of manipulator can ensure the collision safety.

## 5. Discussion and Conclusions

To weaken the impact of nonlinear disturbances on collision detection of sensorless manipulators, a method of disturbance recognition and collision detection based on external torque observer was studied. Regarding the friction as the main disturbance, this research analyzed the friction to establish the mathematical model. Employing the friction value observed by the external torque observer, this research achieved effective parameter identification via the genetic algorithm. Combining theory and experiment, this research arranged multiple sets of experiments to simulate collisions of the manipulator with human in motion and verified the accuracy of the collision detection by the external torque observer after compensating the friction disturbance.

Most of existing sensorless collision detection have complied friction compensation based on dynamic formulas and identified the friction parameters through online or offline solutions, ignoring the remaining nonlinear disturbance factors such as assembly gap and temperature. The dynamics and other parameters of the manipulator must be reidentified when working in different environments. In contrast, the proposed method of establishing a friction-based disturbance model by observing changes on disturbance in this paper can solve the above deficiency. The corresponding disturbance model can be directly transformed according to different working environments. Also, some of the remaining nonlinear disturbance factors are coupled in the identification process, and the identification process is simple and fast.

Overall, this research has successfully provided a low-cost disturbance recognition and collision detection method that can be applied to different working environments and improve the safety of human-robot interaction of cooperative manipulators. The results fully prove the proposed scheme feasible with engineering and theoretical significance. In further research, the disturbance model of the manipulator under variable working conditions will be analyzed and compared in detail.

## Figures and Tables

**Table 1:** Identified parameters of three-joint manipulator friction model.

Joint	$\sigma_{0}$	$\sigma_{1}$	$\sigma_{2}$	$\tau_{c}$	$\tau_{s}$	$\omega_{s}$
1	98,467.9088	314.0483	6.4865	7.8322	4.0033	0.0978
2	95,487.8562	499.4331	6.1838	8.1855	4.5407	0.0996
3	94,546.6673	489.1297	5.3929	7.0023	4.9450	0.0917

**Table 2:** Root mean squared (RMS) error between the calculated friction based on LuGre model and the referred method.

Joint	1	2	3
RMS	0.795	1.02	0.697
